# Muscle layer injury during underwater endoscopic mucosal resection for an adenoma on the cecal fold

**DOI:** 10.1055/a-2447-8225

**Published:** 2024-11-18

**Authors:** Takayuki Nagahashi, Koichi Hamada, Yoshinori Horikawa, Kae Techigawara, Masafumi Ishikawa, Michitaka Honda, Tamotsu Sugai

**Affiliations:** 1 Gastroenterology, Southern Tohoku General Hospital, Koriyama, Japan; 2 12775 Minimally Invasive Surgical and Medical Oncology, Fukushima Medical University, Fukushima, Japan; 3 Surgery, Southern Tohoku General Hospital, Koriyama, Japan; 4 Pathology, Southern Tohoku General Hospital, Koriyama, Japan

Underwater endoscopic mucosal resection (UEMR) differs from conventional EMR in that it does not require local injection; instead, the colon is filled with water, and the tumor is resected 1 . It is characterized by rates of high en bloc, complete, and R0 resection, with a lower recurrence rate than conventional EMR 2 3 . There are few reports of perforation or muscle layer injury caused by UEMR 4 5 . Here, we report a case in which UEMR was performed for an adenoma located on the fold of the cecum, resulting in muscle layer injury ( Video 1 ).

Muscle layer damage associated with underwater endoscopic mucosal resection (UEMR) for an adenoma on the cecal fold.Video 1

An 85-year-old man presented with an 18-mm flat lesion (0-IIa) in the cecum ( Fig. 1 ). A UEMR technique was applied ( Fig. 2 ). The operator did not know that the lesion was on the cecal fold. After resection the inner muscle layer was seen to be injured ( Fig. 3 ); however, no damage to the external muscle layer or perforation was observed. The operator then realized that the lesion had been above the fold. The wound was treated with through-the-scope clips, and complete closure was achieved ( Fig. 4 ). The patient remained asymptomatic, and computed tomography confirmed the absence of free air. The patient was discharged 2 days after the procedure. The pathological diagnosis was an adenoma with a negative vertical margin. The specimen contained a portion of the muscle layer ( Fig. 5 ).

**Fig. 1:**
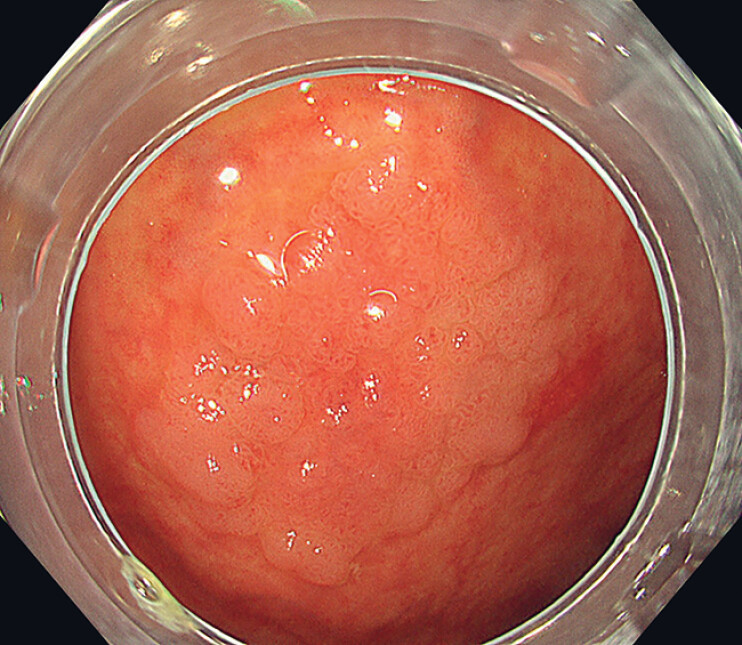
An 18-mm flat lesion (0-IIa) was located on the cecum, in an 85-year-old man. White light image of the lesion.

**Fig. 2:**
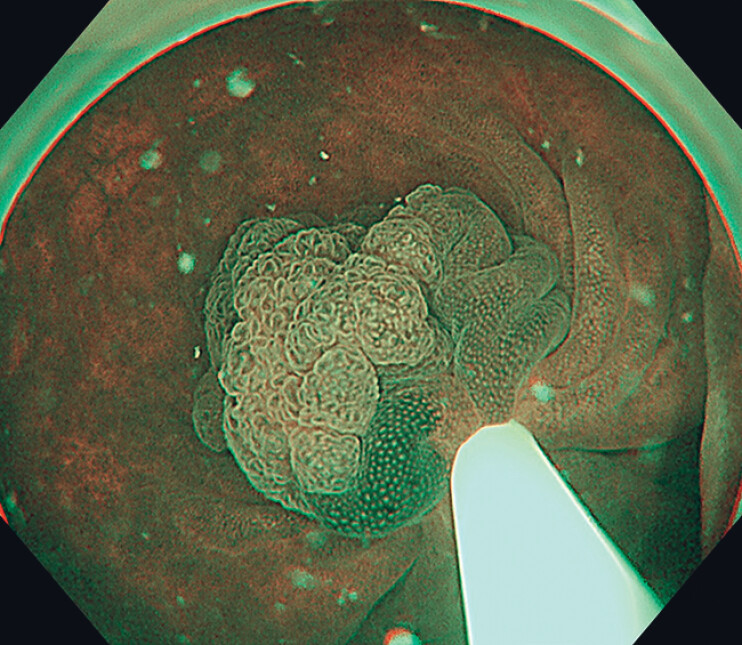
Narrow-band image of the lesion. Underwater endoscopic mucosal resection was performed.

**Fig. 3:**
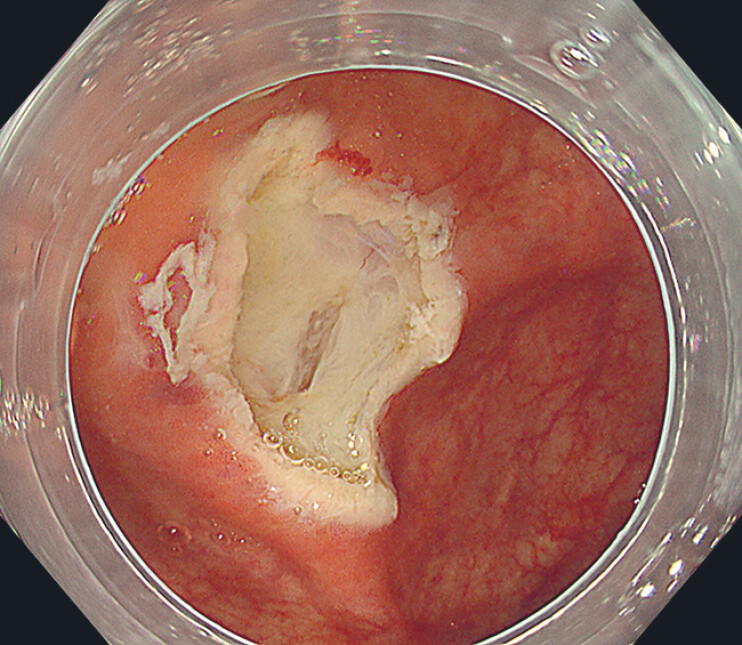
Injury to the muscle layer was seen after resection of the lesion above the cecal fold.

**Fig. 4:**
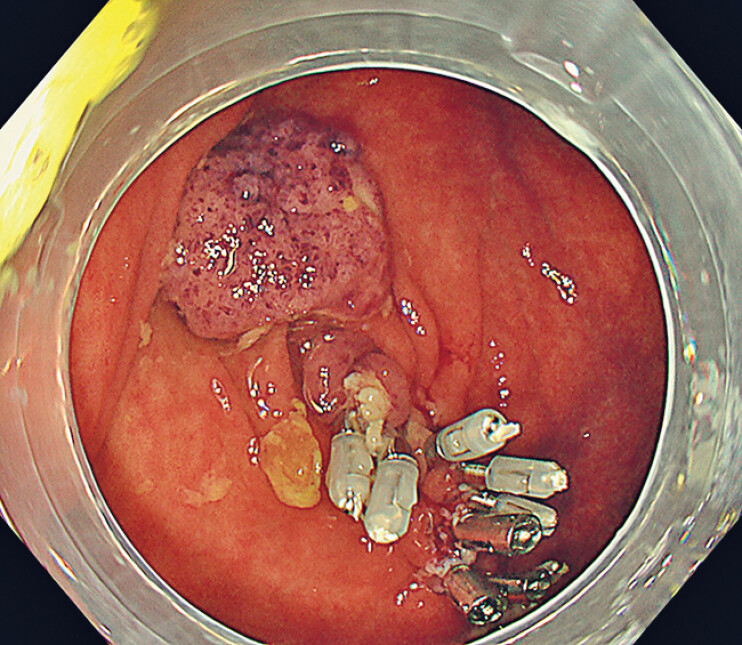
Complete closure was achieved with through-the-scope clips.

**Fig. 5:**
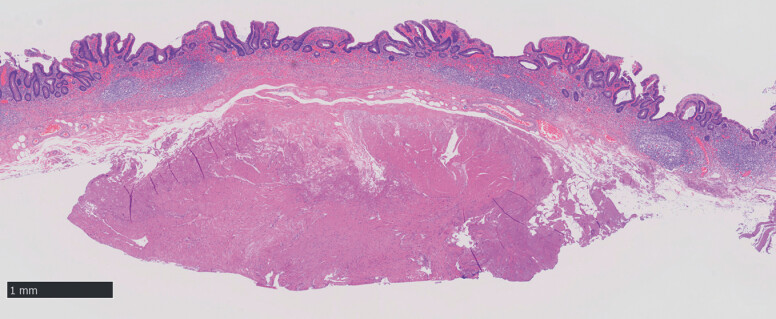
Histopathological appearance of the resected specimen, showing part of the muscle layer.

Several meta-analyses have shown that UEMR has a risk of perforation and bleeding similar to that of conventional EMR 2 3 . However, the factors associated with perforations in UEMR are unknown. In reviewing this case, one possible cause of the muscle injury was the lesion’s location above the fold of the cecum. It may be necessary to inflate the bowel with air before the procedure to ensure that there are no folds under the lesion.

Endoscopy_UCTN_Code_CPL_1AJ_2AD_3AC

## Notes

Endoscopy E-Videos https://eref.thieme.de/e-videos
*E-Videos* is an open access online section of the journal *Endoscopy* , reporting on interesting cases and new techniques in gastroenterological endoscopy. All papers include a high-quality video and are published with a Creative Commons CC-BY license. Endoscopy E-Videos qualify for HINARI discounts and waivers and eligibility is automatically checked during the submission process. We grant 100% waivers to articles whose corresponding authors are based in Group A countries and 50% waivers to those who are based in Group B countries as classified by Research4Life (see: https://www.research4life.org/access/eligibility/ ). This section has its own submission website at https://mc.manuscriptcentral.com/e-videos .
